# Intravenous amino acids to prevent acute kidney injury in cardiac surgery with cardiopulmonary bypass: a meta-analysis of randomized controlled trials^☆^

**DOI:** 10.1016/j.ijcha.2026.101986

**Published:** 2026-08-07

**Authors:** Alexandre N. Pantaleao, Antonio Mutarelli, Jagdip Kang, Marcelo A.P. Braga, Felipe S. Passos, Tulio Caldonazo, Hristo Kirov, Torsten Doenst, Aranya Bagchi, Serguei Melnitchouk

**Affiliations:** a School of Medicine, Federal University of Minas Gerais, Belo Horizonte, Brazil; b Division of Cardiac Surgery, Massachusetts General Hospital, Harvard Medical School, Boston, MA, United States; c Department of Medicine, Federal University of Rio de Janeiro, Rio de Janeiro, Brazil; d Department of Thoracic Surgery, Hospital MaterDei, Salvador, Brazil; e Department of Cardiothoracic Surgery, Friedrich-Schiller-University Jena, Germany; f Department of Anesthesia and Pain Medicine, Massachusetts General Hospital, Harvard Medical School, Boston, MA, United States

**Keywords:** Cardiac surgery, Acute kidney injury, Amino acids, Cardiopulmonary bypass, Perioperative care

## Abstract

**Background:**

The efficacy of intravenous amino acids (AA) in preventing cardiac surgery-associated acute kidney injury (CSA-AKI) remains uncertain.

**Methods:**

Three libraries were searched for randomized controlled trials (RCTs) comparing intravenous AA versus placebo or standard care to reduce the risk of CSA-AKI after cardiac surgery with cardiopulmonary bypass (CPB). The primary endpoint was the incidence of CSA-AKI, overall and stratified by KDIGO stage. Secondary endpoints were need for renal replacement therapy (RRT), 30-day mortality, and 90-day mortality. Risk ratios (RRs) with 95% confidence intervals (CIs) were pooled using a random-effects model.

**Results:**

Five studies were included, comprising 4499 patients, of whom 2247 (50%) were randomized to intravenous AA infusion. Compared with placebo or standard care, intravenous AA administration significantly reduced the risk of CSA-AKI (RR 0.81, 95% CI 0.70–0.94; *p* = 0.005), predominantly driven by one trial. The relative risk reduction was more important for stage 2 or 3 AKI (RR 0.69, 95% CI 0.48–0.99; *p* = 0.046). No significant differences were observed in the need for RRT (*p* = 0.223), 30-day mortality (*p* = 0.893), or 90-day mortality (*p* = 0.581).

**Conclusion:**

Intravenous AA administration is associated with a reduced risk of CSA-AKI across the spectrum of AKI severity, without showing an association with other major clinical outcomes.

## 1 Introduction

Acute kidney injury (AKI) is the most common major complication associated with cardiac surgery [1], affecting 5 to 42% of patients [2]. Cardiac-surgery associated AKI (CSA-AKI) requiring renal replacement therapy (RRT) not only increases hospitalization costs and length of stay in the intensive care unit (ICU), but is also associated with 3–8-fold higher perioperative mortality [3]. Nevertheless, even mild or moderate AKI is associated with increased morbidity and mortality [4]. Despite its prevalence and short- and long-term consequences, there lacks an effective intervention to prevent CSA-AKI, other than supportive measures [5].

The pathophysiology of CSA-AKI is complex, multifactorial, and incompletely understood. Numerous pathways of injury are probably involved, including ischemia-reperfusion injury, inflammation, oxidative stress, and neurohormonal factors [1]. However, it is established that renal hypoperfusion plays a significant role in CSA-AKI, contributing to a decreased glomerular filtration rate after cardiopulmonary bypass (CPB) [6].

Considering the role of hypoperfusion in CSA-AKI, amino acids (AA) infusion emerged as a promising technique to preserve kidney function following cardiac surgery. AA are associated with increased renal plasma flow through decreased afferent arteriole resistance [7], decreased tubule-glomerular feedback activation [8], and increased neuronal nitric oxide synthase in the renal cortex [9]. Beyond biological plausibility, recent studies suggested that intravenous AA may be a pioneer strategy associated with a decreased incidence of CSA-AKI [10], [11], [12], [13], potentially reducing patient morbidity and mortality. However, it remains uncertain role of intravenous AA to prevent CSA-AKI in all its spectrum of severity, as well as other clinical outcomes, specifically in patients undergoing cardiac surgery with CPB.

Therefore, we performed a systematic review and meta-analysis of randomized controlled trials (RCT) examining the efficacy of intravenous AA infusion to prevent CSA-AKI in patients undergoing CPB, specifically interested in AKI incidence and severity, as well as other major clinical endpoints.

## 2 Methods

This systematic review and meta-analysis was performed in line with recommendations from the Cochrane Collaboration and the Preferred Reporting Items for Systematic Reviews and Meta-Analysis (PRISMA) statement guidelines [14]. Accordingly, it was prospectively registered with the International Prospective Register of Systematic Reviews (PROSPERO) under the identification CRD42024584631.

### 2.1 Eligibility criteria

We restricted inclusion in this meta-analysis to studies that examined (i) adults 18 years of age or older, (ii) undergoing cardiac surgery with CPB, (iii) that received intravenous AA. (iv) The study type had to be a RCT and (v) at least one of the following outcomes had to be assessed: AKI incidence, 30 days or 90 days mortality, and incidence of RRT.

We excluded studies that either (i) included patients with preoperative or planned RRT prior to surgery, (ii) administered AA via non-intravenous routes or (iii) administered derivatives of AA. We also excluded conference preprints, conference presentations, protocols, and studies with overlapping populations.

### 2.2 Search strategy and data extraction

We systematically searched three databases (PubMed, Excerpta Medica Database [EMBASE], and the Cochrane Central Register of Controlled Trials) from inception to July 2026 using a combination of subject headings including ‘amino acids’, ‘acute kidney injury’, ‘cardiac surgical procedures’, ‘cardiac surgery’, and ‘bypass’. The entire search string applied to each database is available in the Supplementary Table 1. In addition to searching databases, reference lists of all included studies, meta-analyses and reviews were manually searched to minimize the risk of excluding primary studies that should be included [15].

Two investigators (ANP and AM) independently assessed search results according to predefined criteria to identify eligible studies. The same investigators worked in pair independently to extract key study characteristics and endpoints. Accuracy was verified by a third author (MB). In both instances, disagreements were resolved through consensus.

### 2.3 Study endpoints

Our primary endpoint was the incidence of CSA-AKI (overall and stratified by KDIGO stage) [16], [17]. Prespecified secondary endpoints included (i) need for RRT; (ii) 30-day mortality and (iii) 90-day mortality.

### 2.4 Quality assessment

Two investigators (ANP and MB) independently evaluated the quality of each included article by utilizing the Cochrane tool for assessing the risk of bias in randomized trials (RoB-2) [18]. Accuracy was verified by a third author (AM). Any discrepancies in the quality assessment were resolved through consensus (Supplementary Table 2). We explored the potential for publication bias by visual inspection of the funnel plots for the primary efficacy endpoint.

### 2.5 Statistical analysis

Analyses were conducted according to the intention-to-treat principle. Binary endpoints were compared using Risk Ratios (RRs). All analyses utilized the Mantel-Haenszel random effects model [19] and restricted maximum likelihood (REML) for estimation. Estimates were reported with 95% Confidence Intervals (CIs) and *p*-values, with significance set at *p* < 0.05. Heterogeneity was assessed using the I^2^ statistic, with levels below 40% and Cochran's Q test p-values less than 0.10 indicating negligible heterogeneity, as per the Cochrane Handbook for Systematic Reviews of Interventions [20].

Sensitivity analyses were conducted using a leave-one-out meta-analysis to evaluate the influence of individual studies on the overall effect. Heterogeneity was further explored using Baujat plots [21], and funnel plots were generated to assess the potential for small study effects and publication bias. All statistical analyses were conducted using R version 4.3.0 (R Foundation for Statistical Computing, Vienna, Austria).

## 3 Results

### 3.1 Trial selection and characteristics

**Fig. 1:**
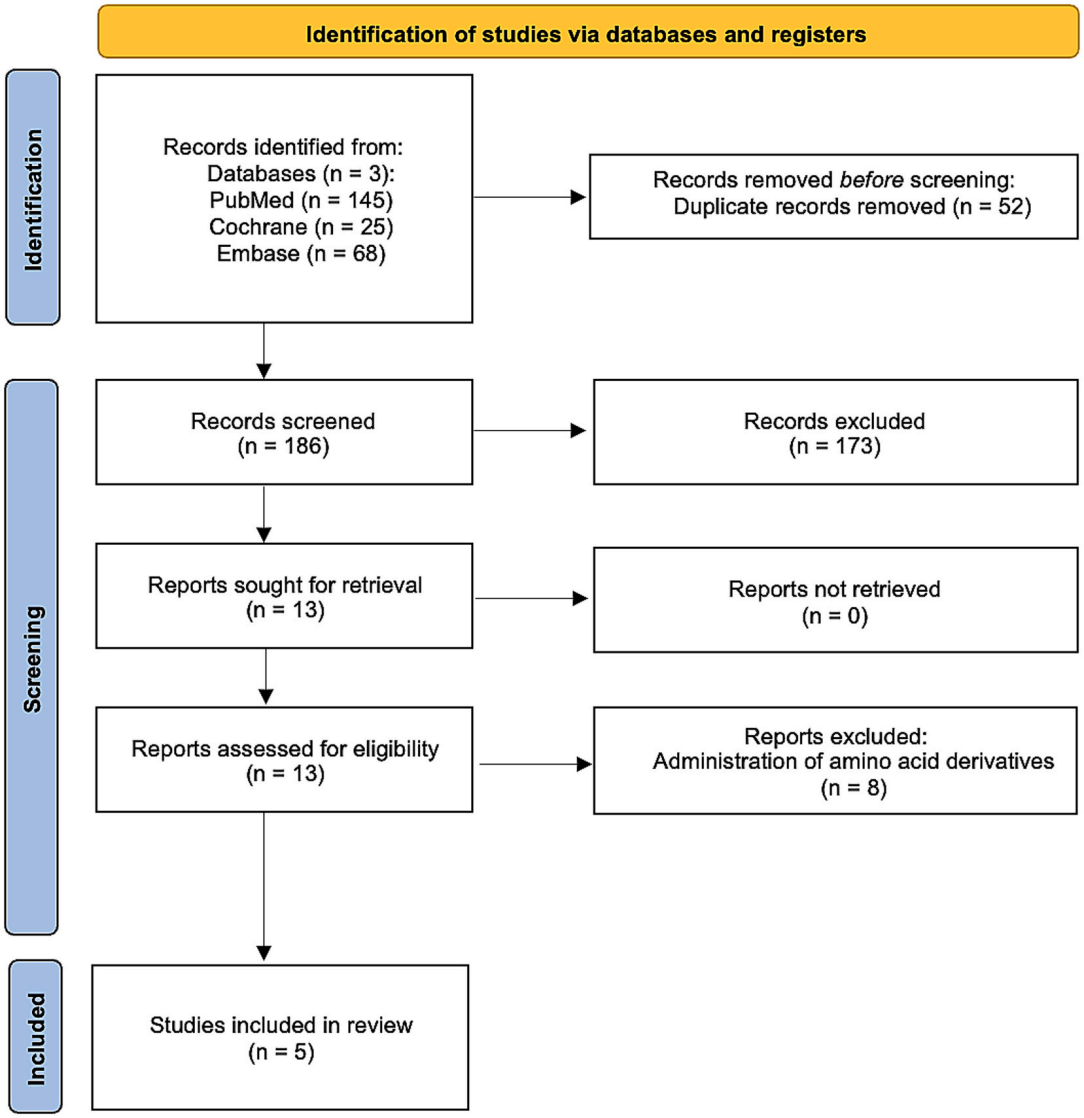
Preferred Reporting Items for Systematic Reviews and Meta-Analyses (PRISMA) flow diagram.

Our systematic search yielded 238 potential articles. After removing duplicate records and studies with an exclusion criterion based on title/abstract review, 13 remained and were thoroughly reviewed for inclusion and exclusion criteria. Ultimately, 5 studies were included [10], [11], [12], [13], [22]. Overall, 4499 patients were studied, of whom 2247 (50%) patients were assigned to intravenous AA infusion and 2252 (50%) were assigned to the control group (standard care or placebo). Comprehensive details of the trial selection are detailed in Fig. 1 and study characteristics are reported in Table 1 and Table 2.

**Table 1:** Baseline characteristics of the included studies.

Trial (year)	Number of patients	Intervention	Age (years)	Female (%)	Cr (mg/dL)	eGFR (mL/min/ 1.73m^2^)	HTN (%)	DM (%)	LVEF (%)	Duration of CPB (min)	Follow-up (days)
	**AA/control**	**AA/control**	**AA/control**	**AA/control**	**AA/control**	**AA/control**	**AA/control**	**AA/control**	**AA/control**	**AA/control**	
Holm (2024)⁎	391/400	L-glutamic acid/placebo	69.3 ± 9.1/ 69.6 ± 9.5	15.9/18.3	1.1 ± 0.3/ 1.1 ± 0.3	75.1 ± 25.5/ 75 ± 27.6	54.3/58.6	0/0	NA	88 ± 35/ 89 ± 37	30
Kazawa (2024)	33/32	Amiparen® / placebo	72(52–77)/ 71(56–75)	34.4/51.5	0.79(0.66–0.93)/0.87 (0.7–1.14)	67(56–75)/ 64(45–80)	72.7/65.6	12.1/9.4	60(60–65)/ 61(59–65)	242 (226–275)/287 (227–338)	90
Landoni (2024)	1759/1752	Isopuramin 10%, Baxter/ placebo	66(57–73)/ 67(58–73)	30.1/30.1	0.96(0.81–1.11)/0.94(0.81–1.1)	78(62–99)/ 77(61–98)	62/61.8	17.6/19.1	59(54–63)/ 60(54–63)	93(73–121)/ 94(73–122)	180
Pu (2019)	33/36	Synthamin 17 Electrolyte Free, Baxter/ standard care	72.3 ± 9.5/ 70.8 ± 9	42.4/13.9	1.14 ± 0.39/ 1.05 ± 0.31	55.7 ± 20.2/ 64.4 ± 18.6	87.9/91.7	21.2/13.9†	56.1 ± 9.5/ 56.9 ± 9.6	248 ± 121/ 286 ± 147	90
Weiss (2022)	31/32	L-Alanyl-Glutamine/ placebo	70.2 ± 10.3/ 66.6 ± 10.5	16.1/15.6	1 ± 0.31/ 0.99 ± 0.2	66.4 ± 17.4/ 72.2 ± 12.3	87.1/78.1	12.9/28.1	NA	117 (94–155)/124 (93–159)	90

Numbers are reported as mean (SD) or median (Q1-Q3), according to the original study. Abbreviations: AA, amino acid; CPB: cardiopulmonary bypass; Cr, creatinine; DM: diabetes mellitus; eGFR: estimated glomerular filtration rate; HTN: hypertension; LVEF: left ventricular ejection fraction.

⁎ This study comprises the non-diabetic patients that required CPB during cardiac surgery from the trials GLUTAMICS I and II.

† Data was available only on diabetes requiring insulin.

**Table 2:** Continuation of characteristics of the included studies.

Trial (year)	Single or multicenter	Population	eGFR to be included (mL/min/ 1.73m^2^)	AKI criteria	Cardiac surgeries analyzed	Timing of AA administration	Timing of AA termination	Total intake of AA (g)
Holm (2024)⁎	Multicenter	Adults <85 years undergoing CABG for acute coronary syndrome with CPB, some at moderate to high risk of postoperative heart failure‡	Not on preoperative dialysis, eGFR not specified	RIFLE	CABG ± valve surgery, others	GLUTAMICS I: After the start of general anesthesia GLUTAMICS II: 10–20 min before reperfusion in CABG	Based on clinical experience	9.2g†
Kazawa (2024)	Single center	>20 years, cardiac surgery requiring CPB, planned to go to ICU post-surgery	≥15	KDIGO	Thoracic aortic replacement or thoracoabdominal aortic replacement	After the start of general anesthesia	72 h	60 (53.3–60)
Landoni (2024)	Multicenter	≥ 18 years, cardiac surgery requiring CPB, expected to stay at the ICU for at least one night	≥30	Creatinine KDIGO§	CABG ± valve surgery, others	Operating room admission	Until 72 h after initiation, discharge from ICU, initiation of RRT, or death (whichever occurred first)	126 (100–259)
Pu (2019)	Single center	Adults, cardiac surgery expected to require at least 1 h of CPB	≥20 and < 90	Creatinine KDIGO§	CABG ± valve surgery, others	After the start of general anesthesia	Until discharge from the ICU/HDU to the ward	22.3 ± 6.9
Weiss (2022)	Single center	18–90 years, cardiac surgery requiring CPB, urinary [TIMP-2]⁎[IGFBP7] ≥0.3 four hours after CPB	≥30	KDIGO	CABG ± valve surgery, others	4 h after terminating CPB	12 h after initiation	0.5 g/kg body weight

Numbers are reported as mean (SD) or median (Q1-Q3), according to the original study. Abbreviations: AA, amino acid; AKI, acute kidney injury; CABG: coronary artery bypass graft; CPB, cardiopulmonary bypass; eGFR: estimated glomerular filtration rate; HDU: high-dependency unit; ICU, intensive care unit; RRT: renal-replacement therapy.

⁎ This study comprises the non-diabetic patients that required CPB during cardiac surgery from the trials GLUTAMICS I and II.

† This was the maximum dose targeted for the patients. Median or mean total intake or AA was not reported.

‡ The risk was only used in the inclusion of patients to GLUTAMICS II trial and was defined as it follows: either a preoperative left ventricular ejection fraction ≤0.30 or a European System for Cardiac Risk Evaluation II (EuroSCORE II) score ≥ 3.0, with at least one of the following cardiac or procedure- related risk factors: an urgent or emergency procedure, Canadian Cardiovascular Society class IV angina, left ventricular ejection fraction ≤0.50, myocardial infarction within 90 days prior, or a concomitant aortic or mitral valve procedure.

§ In these studies, urinary output from KDIGO was not considered a criterion for AKI.

### 3.2 Primary endpoint

**Fig. 2:**
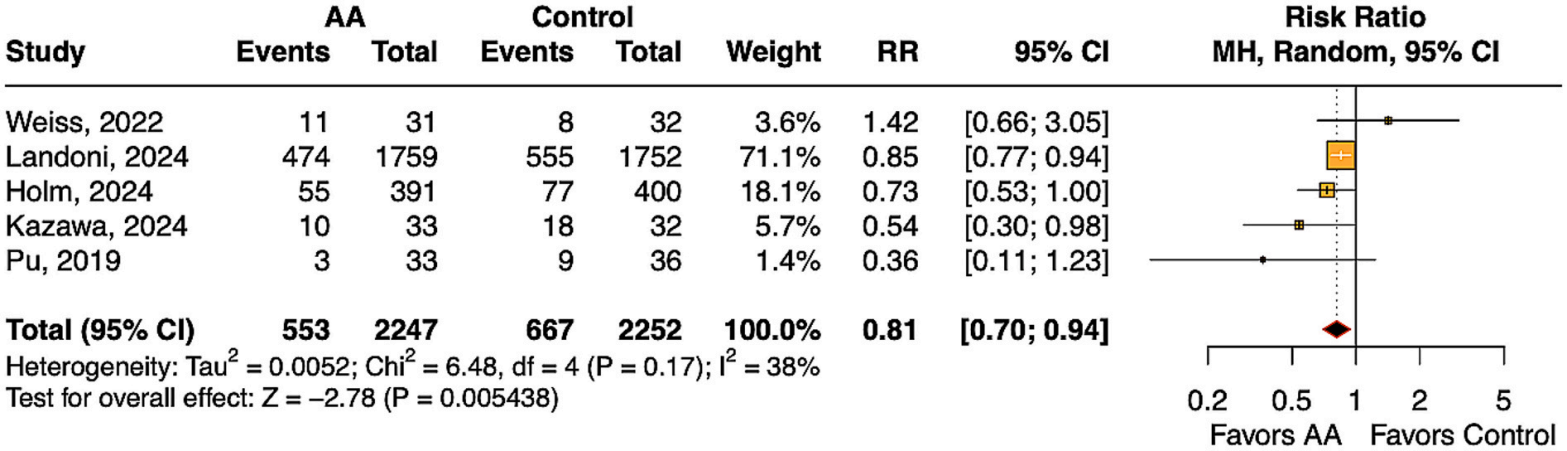
Forest plot for the incidence of overall CSA-AKI. AA: amino acids, CSA-AKI: cardiac-surgery associated acute kidney injury, CI: confidence interval, MH: Mantel-Haenszel, RR = risk ratio.

**Fig. 3:**
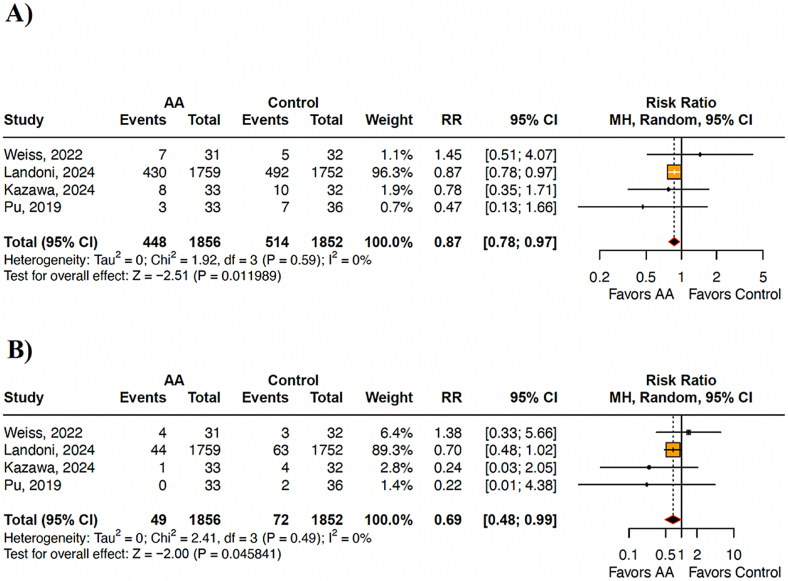
Forest plot for the incidence of CSA-AKI stratified by KDIGO stage 1 (A) and stage 2 or 3 (B). AA: amino acids, CSA-AKI: cardiac-surgery associated acute kidney injury, CI: confidence interval, MH: Mantel-Haenszel, RR = risk ratio.

Intravenous AA infusion was associated with a significantly reduced risk of CSA-AKI compared to placebo or standard care (RR 0.81, 95% CI 0.70–0.94, *p* = 0.005, I^2^ = 38%, Fig. 2), predominantly driven by the PROTECTION trial [10]. The protective effect was also observed in preventing stage 1 (RR 0.87, 95% CI 0.78–0.97, *p* = 0.012, I^2^ = 0%, Fig. 3A), and stage 2 or 3 AKI (RR 0.69, 95% CI 0.48–0.99, *p* = 0.046, I^2^ = 0%, Fig. 3B).

**Fig. 4:**
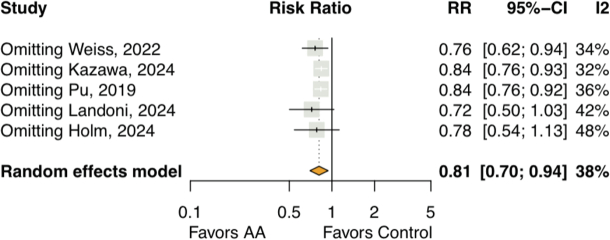
Leave-one-out analysis for the primary outcome (incidence of CSA-AKI). AA: amino acids, CSA-AKI: cardiac-surgery associated acute kidney injury, CI: confidence interval, MH: Mantel-Haenszel, RR = risk ratio.

Regarding overall incidence of CSA-AKI, the leave-one-out analysis indicated that the protective effect remained significative with the individual exclusion of most trials (Fig. 4). However, the exclusion of the PROTECTION trial by Landoni et al. [10] yielded a non-significant result (RR 0.72, 95% CI 0.50–1.03, I^2^ = 42%), as well as the exclusion of the trial by Holm et al. [11] (RR 0.78, 95% CI 0.54–1.13, I^2^ = 48%).

When dividing the studies according to the intervention (mixture AA solution vs single AA administration), no significant effect was observed for the primary endpoint in both subgroups (RR 0.93, 95% CI 0.50–1.73, I^2^ = 59.5%, *p* = 0.12 for single AA and RR 0.68, 95% CI 0.44–1.06, I^2^ = 49.4%, *p* = 0.14 for mixture AA solutions, Supplementary Fig. 1).

While the trials by Weiss and Kazawa [13], [22] applied the complete KDIGO criteria for diagnosing AKI, Landoni and Pu [10], [12] applied only the creatinine criteria from KDIGO and Holm [11] used the RIFLE criteria. A subanalysis comprising only the trials that used the complete KDIGO criteria also resulted in a non-significant effect for the primary endpoint (RR 0.85, 95% CI 0.33–2.19, I^2^ = 73.7%, *p* = 0.051, Supplementary Fig. 2). Creatinine-only studies likely underestimate the true incidence of AKI and this analysis applying the full KDIGO criteria, although underpowered, might reflect a more accurate and less overestimated effect of AA to prevent CSA-AKI.

Visual inspection of the funnel plot for the primary efficacy endpoint did not suggest evidence of publication bias (Supplementary Fig. 3). The Baujat plot suggests that the studies that contributed the most for the heterogeneity did not have an important influence on the overall effect (Supplementary Fig. 4).

### 3.3 Secondary endpoints

**Fig. 5:**
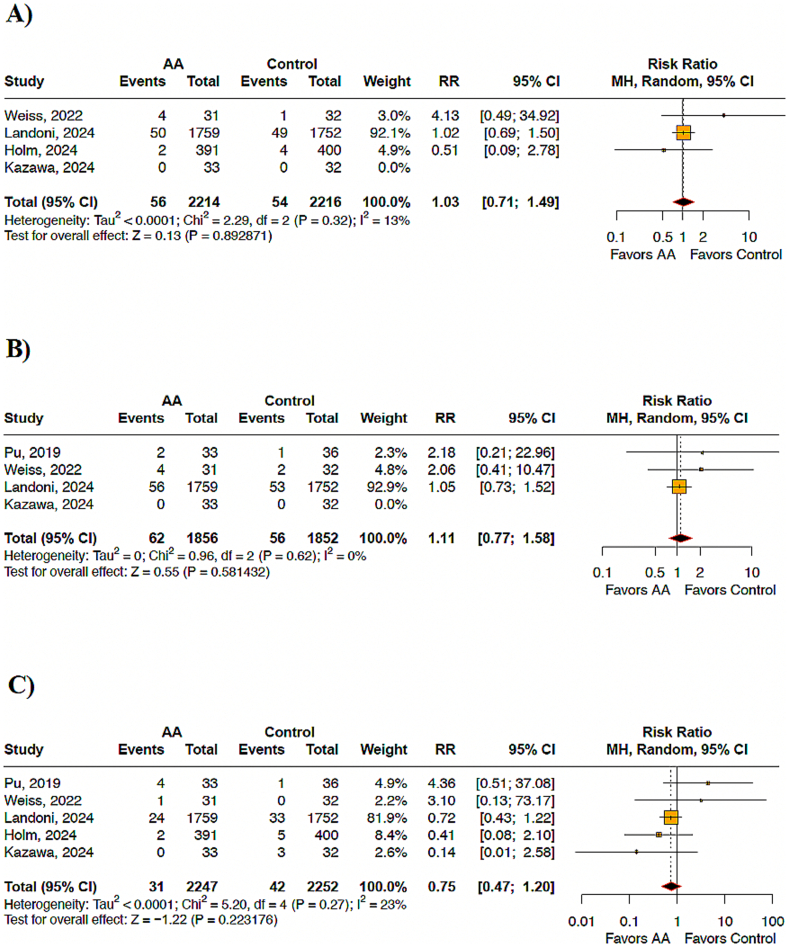
Forest plot for mortality in 30 days (A), 90 days (B) and need for renal replacement therapy (C). AA: amino acids, CSA-AKI: cardiac-surgery associated acute kidney injury, CI: confidence interval, MH: Mantel-Haenszel, RR = risk ratio.

There was no difference regarding 30-day mortality (RR 1.03, 95% CI 0.71–1.49, *p* = 0.893, I^2^ = 13%, Fig. 5A), 90-day mortality (RR 1.11, 95% CI 0.77–1.58, *p* = 0.581, I^2^ = 0%, Fig. 5B), and need for RRT (RR 0.75, 95% CI 0.47–1.20, *p* = 0.223, I^2^ = 23%, Fig. 5C).

**Risk of Bias.**

Among the five studies, four of them had low concerns in all five domains of the ROB-2 Cochrane Collaboration's tool (Supplementary Table 2). However, the trial by Holm et al. [11] had some concerns regarding the selection of the reported results, once that the trials were not initially designed to have AKI as the primary outcome. Post-hoc analysis potentially led to an overestimated effect and therefore could influence the interpretation of pooled results.

## 4 Discussion

This comprehensive meta-analysis of 5 studies and 4499 patients assessed the efficacy of intravenous AA administration to prevent CSA-AKI in patients undergoing CPB. The main findings were that intravenous AA were associated with reduction of the incidence of CSA-AKI (overall and stratified by KDIGO stage) without showing significantly difference of need for RRT or postoperative mortality.

Besides supportive measures, no single strategy is definitively proven to be effective in preventing CSA-AKI. Numerous studied interventions, such as nitric oxide and fenoldopam, directly target renal hypoperfusion. Despite the vasodilator effect and theoretical benefit, mixed results in the literature [23], [24], [25] hinder the recommendation of these strategies to prevent CSA-AKI. Different medications targeting diverse pathophysiological mechanisms have also been proposed to prevent CSA-AKI, including antioxidant, anti-inflammatory and statins therapy. Yet, none shows consistent results supporting their use [26]. Some therapies, such as haptoglobin and iron chelators, have demonstrated a potential benefit in CSA-AKI in animal studies [27], [28], but further clinical studies are needed to investigate their true efficacy. Our data support the emergence of AA as a potentially effective strategy to prevent CSA-AKI.

A recent study conducted by Martín-Fernandez et al. [26] provided a pan-comparative meta-analysis of more than seven strategies and medications to prevent CSA-AKI, and found that none provides solid and significant protection against CSA-AKI. In their analysis, the studies of Pu et al. [12], and Holm et al. [11] were also included. However, these interventions were classified as ‘others’ in the subgroup analysis due to the lack of other studies with AA administration. The RCTs of Weiss et al. [22], Landoni et al. [10] and Kazawa et al. [13] provided data on additional 3640 patients treated with either intravenous AA or placebo, and are now investigated by our present meta-analysis.

In the setting of CPB, renal hypoperfusion is an essential contributor to CSA-AKI [29], and AA might attenuate this injury through increased renal plasma flow [10]. This effect is mediated chiefly by decreased afferent arteriole resistance [7], decreased tubule-glomerular feedback activation [8], and increased neuronal nitric oxide synthase in the renal cortex [9]. Additionally, murine studies demonstrated that glutamine attenuates kidney damage by downregulating proapoptotic pathways in tubular epithelial cells [30]. Glutamate may also prevent renal hypoperfusion through improved recovery of cardiac function after ischemia [31], [32]. Our meta-analysis combining the effects of single AA and mixture of AA administrations revealed the protective effect of intravenous AA administration in CSA-AKI in the context of CPB.

A meta-analysis conducted by Pruna et al. [33] investigated the effect of perioperative intravenous AA solutions in kidney protection. This study included the trials of Landoni et al. [10], Pu et al. [12], and Kazawa et al. [13], and found no association between intravenous AA solutions and CSA-AKI using a random-effects model. Nevertheless, our study included two additional RCTs that administered single-AA infusions [11], [22], resulting in 854 more patients, and we identified the protective effect of intravenous AA in CSA-AKI. Furthermore, the mentioned study did not perform analysis focused on cardiac surgery beyond AKI incidence. As CPB plays an essential role in CSA-AKI, extrapolating data from different surgeries that do not require CPB may not be accurate.

In addition, another recent meta-analysis led by Jiang et al. [34] examined the protective effect of intravenous AA solutions on kidney function. This work included the studies analyzed in our meta-analysis and also revealed a protective effect of intravenous AA administration in preventing CSA-AKI. However, similarly to the study conducted by Pruna et al. [33], there were no further analysis focusing solely on patients undergoing cardiac surgery, once that the work by Jiang et al. [34] also analyzed studies with ICU patients that did not undergo cardiovascular surgery. A secondary analysis of the PROTECTION trial revealed that patients with prolonged CPB exposure had a higher incidence of CSA-AKI and benefited more from intravenous AA [35]. Our analysis of incidence of AKI stratified by severity, need for RRT and postoperative mortality provide important and novel insights to guide the care of patients undergoing cardiac surgery with CPB.

### 4.1 Study strengths and limitations

This meta-analysis of RCTs addressed this important topic in cardiac surgery that may improve the management of patients undergoing cardiac surgery with CPB. Besides CSA-AKI, other three outcomes were addressed. However, this work has the intrinsic limitations of a study level meta-analysis, including the risk of methodological heterogeneity of the included studies and residual confounders. The significant heterogeneity in studies regarding procedures, AA solutions, timings and dosages limit the robustness of our analysis. While four trials initiated AA perioperatively, Weiss et al. [22] initiated 4 h after terminating CPB, and this difference may result in effect modification. A secondary analysis of the PROTECTION trial found that brief (≤48 h) AA infusion revealed lower ICU mortality compared to prolonged (>48 h and ≤ 72 h). Therefore, while definitive evidence is pending, restricting AA infusion to ≤48 h should be considered [36], [37].

Moreover, the absence of patient-level data limits a more complete report on the subgroups undergoing cardiac surgery. The largest trial included, Landoni et al. [10], had a notable influence in the overall result as it contributed for most of the sample size in the analysis. Nonetheless, the inclusion of other smaller but similar studies from different sites and investigators increases the validity and reproducibility of the results.

There was notable heterogeneity specifically regarding the AA administered in each trial. However, the mentioned biological basis behind the administration of AA solutions or isolated AA supports the combination of these studies in our analysis. Furthermore, there was no statistical heterogeneity in the analyzed outcomes and sensitivity analysis reinforced the potentially negligible influence of heterogeneity on the overall effect. The criteria defining AKI also varied among the studies (two applied KDIGO criteria, two employed only creatinine from KDIGO and one used RIFLE criteria). Although the standardization of KDIGO criteria including urinary output would potentially increase the incidence of CSA-AKI, the criteria used are fundamentally similar and comparable, therefore this should not substantially impact the outcomes. The exclusion of diabetic patients in the trial conducted by Holm et al. [11] could underestimate the effect of AA, but it should not importantly affect the interpretation of outcomes, as all other studies included this population, and the sensitivity analysis raised no concern for the effect of heterogeneity on the overall effect. In addition, a secondary analysis of the PROTECTION trial showed similar effectiveness of perioperative AA infusion among patients with or without diabetes [38]. Last, despite lacking peer review, the exclusion of conference abstracts and preprints could introduce publication bias to our results.

## 5 Conclusion

The present meta-analysis shows that intravenous AA administration is associated with reduced risk of CSA-AKI across the entire spectrum of AKI severity, without showing association with other major clinical outcomes.

## CRediT authorship contribution statement

**Alexandre N. Pantaleao:** Writing – original draft, Methodology, Investigation, Conceptualization. **Antonio Mutarelli:** Investigation, Formal analysis. **Jagdip Kang:** Writing – review & editing, Supervision. **Marcelo A.P. Braga:** Software, Investigation, Formal analysis, Data curation. **Felipe S. Passos:** Writing – review & editing, Validation. **Tulio Caldonazo:** Writing – review & editing, Validation, Project administration, Funding acquisition. **Hristo Kirov:** Writing – review & editing, Supervision. **Torsten Doenst:** Writing – review & editing, Validation. **Aranya Bagchi:** Writing – review & editing, Visualization, Supervision. **Serguei Melnitchouk:** Writing – review & editing, Validation, Supervision.

## Declaration of competing interest

The authors declare that they have no known competing financial interests or personal relationships that could have appeared to influence the work reported in this paper.

## References

[1] Wang Y., Bellomo R. (2017). Cardiac surgery-associated acute kidney injury: risk factors, pathophysiology and treatment. Nat. Rev. Nephrol..

[2] Ostermann M., Kunst G., Baker E., Weerapolchai K., Lumlertgul N. (2021). Cardiac surgery associated AKI prevention strategies and medical treatment for CSA-AKI. J. Clin. Med..

[3] Ortega-Loubon C., Fernández-Molina M., Carrascal-Hinojal Y., Fulquet-Carreras E. (2016). Cardiac surgery-associated acute kidney injury. Ann. Card. Anaesth..

[4] Boyer N., Eldridge J., Prowle J.R., Forni L.G. (2022). Postoperative acute kidney injury. Clin. J. Am. Soc. Nephrol..

[5] Zarbock A., Küllmar M., Ostermann M., Lucchese G., Baig K., Cennamo A. (2021). Prevention of cardiac surgery-associated acute kidney injury by implementing the KDIGO guidelines in high-risk patients identified by biomarkers: the PrevAKI-Multicenter randomized controlled trial. Anesth. Analg..

[6] Meersch M., Volmering S., Zarbock A. (2017). Prevention of acute kidney injury. Best Pract. Res. Clin. Anaesthesiol..

[7] Meyer T.W., Ichikawa I., Zatz R., Brenner B.M. (1983). The renal hemodynamic response to amino acid infusion in the rat. Trans. Assoc. Am. Phys..

[8] Seney F.D., Persson E.G., Wright F.S. (1987). Modification of tubuloglomerular feedback signal by dietary protein. Am. J. Phys..

[9] Tolins J.P., Shultz P.J., Westberg G., Raij L. (1995). Renal hemodynamic effects of dietary protein in the rat: role of nitric oxide. J. Lab. Clin. Med..

[10] Landoni G., Monaco F., Ti L.K., Redaelli M.B., Bradic N., Comis M. (2024). A randomized trial of intravenous amino acids for kidney protection. N. Engl. J. Med..

[11] Holm J., Vanky F., Svedjeholm R. (2024). Association of Glutamate Infusion with Risk of acute kidney injury after coronary artery bypass surgery: a pooled analysis of 2 randomized clinical trials. JAMA Netw. Open.

[12] Pu H., Doig G., Heighes P., Allingstrup M., Wang A., Brereton J. (2019). Intravenous amino acid therapy for kidney protection in cardiac surgery patients: a pilot randomized controlled trial. J. Thorac. Cardiovasc. Surg..

[13] Kazawa M., Kabata D., Yoshida H., Minami K., Maeda T., Yoshitani K. (2024). Amino acids to prevent cardiac surgery-associated acute kidney injury: a randomized controlled trial. JA Clin. Rep..

[14] Moher D., Liberati A., Tetzlaff J., Altman D.G., PRISMA Group (2009). Preferred reporting items for systematic reviews and meta-analyses: the PRISMA statement. PLoS Med..

[15] Wohlin C., Kalinowski M., Romero Felizardo K., Mendes E. (2022). Successful combination of database search and snowballing for identification of primary studies in systematic literature studies. Inf. Softw. Technol..

[16] Bellomo R, Ronco C, Kellum JA, Mehta RL, Palevsky P, Acute Dialysis Quality Initiative workgroup. Acute renal failure - definition, outcome measures, animal models, fluid therapy and information technology needs: the second international consensus conference of the Acute Dialysis Quality Initiative (ADQI) Group. Crit. Care 2004; 8(4):R204–R212. doi:10.1186/cc2872 PubMed PMID: 15312219; PubMed Central PMCID: PMC522841. PMC522841 15312219

[17] Notice (2012). Kidney Int. Suppl..

[18] Higgins J.P.T., Altman D.G., Gøtzsche P.C., Jüni P., Moher D., Oxman A.D. (2011). The Cochrane collaboration’s tool for assessing risk of bias in randomised trials. BMJ.

[19] Mantel N., Haenszel W. (1959). Statistical aspects of the analysis of data from retrospective studies of disease. J. Natl. Cancer Inst..

[20] (2026). Chapter 10: Analysing data and undertaking meta-analyses | Cochrane Training [Internet].

[21] Baujat B., Mahé C., Pignon J.P., Hill C. (2002). A graphical method for exploring heterogeneity in meta-analyses: application to a meta-analysis of 65 trials. Stat. Med..

[22] Weiss R., Von Groote T., Meersch M., Schafers M., Kellum J., Zarbock A. (2023). The effect of glutamine on kidney damage in cardiac surgery patients at high risk for AKI: a double-blind randomized controlled trial. Critical care (London, England)..

[23] Vuong T.A., Rana M.S., Moore B., Cronin J., Ceneri N.M., Sinha P. (2022). Association between exogenous nitric oxide given during cardiopulmonary bypass and the incidence of postoperative kidney injury in children. J. Cardiothorac. Vasc. Anesth..

[24] Lei C., Berra L., Rezoagli E., Yu B., Dong H., Yu S. (2018). Nitric oxide decreases acute kidney injury and stage 3 chronic kidney disease after cardiac surgery. Am. J. Respir. Crit. Care Med..

[25] Kamenshchikov N.O., Anfinogenova Y.J., Kozlov B.N., Svirko Y.S., Pekarskiy S.E., Evtushenko V.V. (2022). Nitric oxide delivery during cardiopulmonary bypass reduces acute kidney injury: a randomized trial. J. Thorac. Cardiovasc. Surg..

[26] Martín-Fernández M., Casanova A.G., Jorge-Monjas P., Morales A.I., Tamayo E., López Hernández F.J. (2024). A wide scope, pan-comparative, systematic meta-analysis of the efficacy of prophylactic strategies for cardiac surgery-associated acute kidney injury. Biomed. Pharmacother..

[27] Sharma S., Leaf D.E. (2019). Iron chelation as a potential therapeutic strategy for AKI prevention. J. Am. Soc. Nephrol..

[28] Kubota K., Egi M., Mizobuchi S. (2017). Haptoglobin Administration in Cardiovascular Surgery Patients: its association with the risk of postoperative acute kidney injury. Anesth. Analg..

[29] Yang X, Zhu L, Pan H, Yang Y. Cardiopulmonary bypass associated acute kidney injury: better understanding and better prevention. Ren. Fail. 46(1):2331062. doi:10.1080/0886022X.2024.2331062 PubMed PMID: 38515271; PubMed Central PMCID: PMC10962309. PMC10962309 38515271

[30] Thomas K., Zondler L., Ludwig N., Kardell M., Lüneburg C., Henke K. (2022). Glutamine prevents acute kidney injury by modulating oxidative stress and apoptosis in tubular epithelial cells. JCI Insight.

[31] Burns A.H., Reddy W.J. (1978). Amino acid stimulation of oxygen and substrate utilization by cardiac myocytes. Am. J. Phys..

[32] Svedjeholm R., Vanhanen I., Håkanson E., Joachimsson P.O., Jorfeldt L., Nilsson L. (1996). Metabolic and hemodynamic effects of intravenous glutamate infusion early after coronary operations. J. Thorac. Cardiovasc. Surg..

[33] Pruna A., Losiggio R., Landoni G., Kotani Y., Redaelli M.B., Veneziano M. (2024). Amino acid infusion for perioperative functional renal protection: a meta-analysis. J. Cardiothorac. Vasc. Anesth..

[34] Jiang W., Shi K., Shao J., Song L., Shi Y., Wang H. (2025). Protective effect of intravenous amino acid on kidney function: a systematic review and meta-analysis of randomized controlled trials. J. Crit. Care.

[35] Pontillo D., Rong L.Q., Pruna A., Pisano A., Monaco F., Bruni A. (2025). Impact of cardiopulmonary bypass duration on the renal effects of amino acids infusion in cardiac surgery patients. J. Cardiothorac. Vasc. Anesth..

[36] Landoni G., Oriani A., Ti L.K., Losiggio R., Bradic N., Pruna A. (2026). Association of duration of amino acids infusion and renal protection: a secondary analysis of the PROTECTION trial. Br. J. Anaesth..

[37] Stoppe C. (2026). Timing matters: the delicate balance in amino acids infusion duration in cardiac surgery. Br. J. Anaesth..

[38] Consonni M., Fresilli S., Kotani Y., Garofalo E., Bradic N., Scandroglio A.M. (2026). Diabetes does not modify the renal-protective effect of intravenous amino acids infusion after cardiac surgery. J. Endocrinol. Investig..

