# Supplementary material for: Intravenous amino acids to prevent acute kidney injury in cardiac surgery with cardiopulmonary bypass: a meta-analysis of randomized controlled trials
Source: Int J Cardiol Heart Vasc. 2026 Aug 7;66:101986. doi: 10.1016/j.ijcha.2026.101986 (PMC13476533; doi:10.1016/j.ijcha.2026.101986)
Supplement: Supplementary file 2 [file mmc2.docx]

**Supplementary Material**

Supplementary Table 1. Complete search strategy.

Supplementary Table 2. Critical appraisal according to the ROB-2 Cochrane Collaboration’s tool for assessing risk of bias in randomized trials.

Supplementary Figure 1: Forest plot for the incidence of CSA-AKI dividing the studies into single versus mixture amino acid solutions

Supplementary Figure 2: Forest plot for the incidence of CSA-AKI comprising the studies that used complete KDIGO criteria

Supplementary Figure 3: Funnel plot for the primary endpoint (incidence of CSA-AKI).

Supplementary Figure 4: Baujat plot for the primary endpoint (incidence of CSA-AKI).

**Supplementary Table 1.** Complete search strategy.

Search string, July 2026:

**PubMed:** ("Amino Acids" [Mesh] OR "Amino Acid*" OR "acidic aminoacid" OR ("amino acid" AND ("residue" OR "solution" OR "basic" OR "cyclic" OR "acidic")) OR "free amino acid" OR "free aminoacid" OR "l amino acid" OR "l aminoacid") AND ("Acute Kidney Injury" [Mesh] OR "AKI" OR "acute kidney insufficiency" OR "acute kidney failure" OR ("acute renal" AND ("failure" OR "insufficiency" OR "Injury" OR "Injuries"))) AND ("Cardiac Surgical Procedures" [Mesh] OR "cardiac surgery" OR "bypass" OR "Heart Bypass, Right" [Mesh] OR "Heart Bypass, Left" [Mesh] OR "cardiosurgery" OR "heart operation" OR "heart surgery")

**EMBASE**: ('amino acid*' OR 'acidic aminoacid' OR ('amino acid' AND ('residue' OR 'solution' OR 'basic' OR 'cyclic' OR 'acidic')) OR 'free amino acid' OR 'free aminoacid' OR 'l amino acid' OR 'l aminoacid') AND ('acute kidney injury' OR 'aki' OR 'acute kidney insufficiency' OR ('acute renal' AND ('failure' OR 'insufficiency' OR 'injury' OR 'injuries')) OR 'acute kidney failure') AND ('cardiac surgical procedures' OR 'cardiac surgery' OR 'bypass' OR 'heart bypass, right' OR 'heart bypass, left' OR 'cardiosurgery' OR 'heart operation' OR 'heart surgery')

**Cochrane:** ("amino acid*" OR "acidic aminoacid" OR "free amino acid" OR "l amino acid") AND ("Acute Kidney Injury" OR "AKI" OR "acute kidney insufficiency" OR ("acute renal" AND ("failure" OR "insufficiency" OR "Injury" OR "Injuries")) OR "acute kidney failure") AND ("Cardiac Surgical Procedures" OR "cardiac surgery" OR "bypass" OR "Heart Bypass, Right" OR "Heart Bypass, Left" OR "cardiosurgery" OR "heart operation" OR "heart surgery")

**Supplementary Table 2.** Critical appraisal according to the ROB-2 Cochrane Collaboration’s tool for assessing risk of bias in randomized trials.

| **Study** | **D1** | **D2** | **D3** | **D4** | **D5** |
| --- | --- | --- | --- | --- | --- |
| Holm (2024) | Low | Low | Low | Low | Some concerns |
| Kazawa (2024) | Low | Low | Low | Low | Low |
| Landoni (2024) | Low | Low | Low | Low | Low |
| Pu (2019) | Low | Low | Low | Low | Low |
| Weiss (2022) | Low | Low | Low | Low | Low |

D1: bias arising from the randomization process; D2: bias due to deviations from intended intervention; D3: bias due to missing outcome data; D4: bias in measurement of the outcome; D5: bias in selection of the reported results

**Supplementary Figure 1:** Forest plot for the incidence of CSA-AKI dividing the studies into single versus mixture amino acid solutions

**Supplementary Figure 2:** Forest plot for the incidence of CSA-AKI comprising the studies that used complete KDIGO criteria

**Supplementary Figure 3**: Funnel plot for the primary endpoint (incidence of CSA-AKI).

**Supplementary Figure 4**: Baujat plot for the primary endpoint (incidence of CSA-AKI).
